# Supplementary material for: Optimizing blood culture diagnostics through laboratory automation: reducing turnaround time and improving clinical outcomes
Source: Microbiol Spectr. 2025 Nov 11;13(12):e01927-25. doi: 10.1128/spectrum.01927-25 (PMC12671119; doi:10.1128/spectrum.01927-25)
Supplement: Fig. S1 — Turnaround times for blood cultures across different regions in mainland China. [file spectrum.01927-25-s0001.docx]

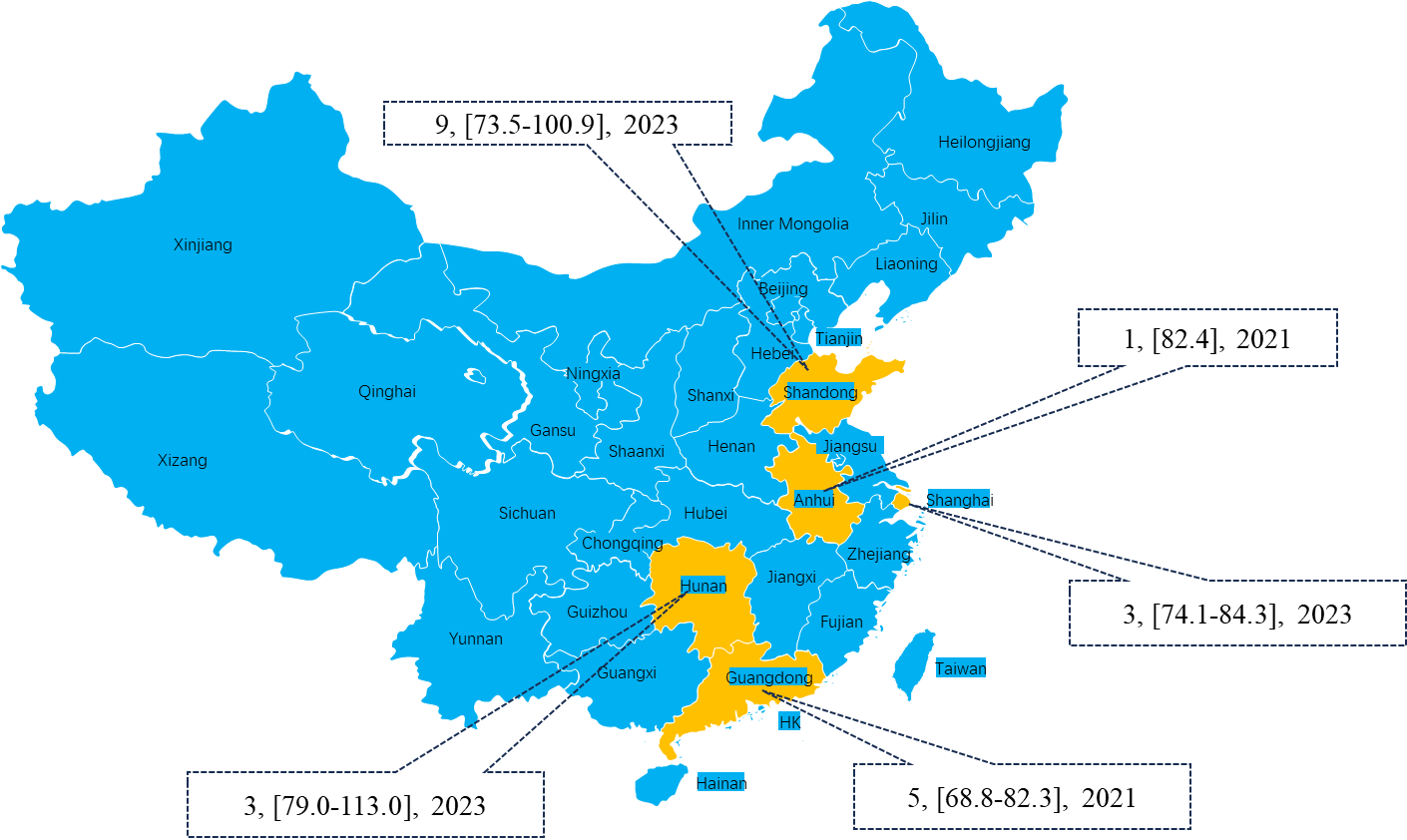


Figure S1. Turnaround times (TAT) for blood cultures across different regions in mainland China. This retrospective multicenter study included 18 hospitals from Shandong, Hunan, Anhui, Guangdong, and Shanghai. For each province, the figure presents the number of hospitals, TAT range, and the corresponding year of data collection.
